# Comparing demersal megafaunal species diversity along the depth gradient within the South Aegean and Cretan Seas (Eastern Mediterranean)

**DOI:** 10.1371/journal.pone.0184241

**Published:** 2017-09-05

**Authors:** Panagiota Peristeraki, George Tserpes, Nikolaos Lampadariou, Kostantinos I. Stergiou

**Affiliations:** 1 Institute of Marine Biological Resources and Inland Waters, Hellenic Centre for Marine Research, Heraklion, Crete, Greece; 2 Biology Department, University of Crete, Heraklion, Crete, Greece; 3 Institute of Oceanography, Hellenic Centre for Marine Research, Heraklion, Crete, Greece; 4 Institute of Marine Biological Resources and Inland Waters, Hellenic Centre for Marine Research, Aghios Kosmas, Athens, Greece; 5 Laboratory of Ichthyology, Department of Zoology, School of Biology, Aristotle University of Thessaloniki, Thessaloniki, Greece; Universita degli Studi di Genova, ITALY

## Abstract

Knowledge on biodiversity patterns of demersal megafaunal species in the Mediterranean and particularly in its eastern basin is still very scarce. In the present study, fine-scale diversity patterns in relation to depth were analyzed for three major megafaunal groups (fish, cephalopods and crustaceans) in three subareas of the eastern Mediterranean (Crete, Cyclades and Dodecanese islands). The analysis was based on data from the Mediterranean International Trawl Survey conducted during 2005–2014 and the relationship between depth and two different diversity measures (species richness and Shannon-Weaver) was examined using Generalized Additive Modeling (GAM) techniques. Species richness of fish decreased with depth in two of the three subareas (Cyclades, Dodecanese), while the opposite was true for crustaceans in all subareas. Cephalopods had higher species richness at intermediate depths, near the shelf break. Significant differences among subareas were found, with Crete showing a distinct species richness-depth pattern, which was more obvious for fish and cephalopods. The differences among subareas were also highlighted based on the occurrence of alien species of Indo-Pacific origin, which were more frequent in Crete. Our results suggested that the importance of depth-related factors in structuring communities was higher for cephalopods and less important for fish, and that Crete showed a distinct diversity-depth relationship, a fact that can be attributed to its specific geographical and oceanographic characteristics. These results support the current GFCM/FAO’s characterization of Crete as a unique geographic subarea. The findings of the study contribute to understanding the causes of underlying diversity patterns and would assist various environmental management actions, particularly those related to the establishment of marine-protected areas.

## Introduction

Species diversity has important implications for the functioning of ecosystems. Understanding the causes of underlying diversity patterns, as well as their interaction with environmental factors, is important for ecosystem conservation purposes.

In the past, numerous publications on aquatic sciences, in different geographic areas, have studied faunal zonation, including the distribution of species along environmental gradients. These studies suggest that physical factors, such as depth, temperature, pressure, hydrographic conditions, oxygen content, sediment type, water mass structure and topography, as well as biological interactions, are possible causes of spatial community changes in marine environments [1–7]. Fishing pressure has been also suggested as a structuring factor for marine communities [8–12]. Among abiotic parameters, depth is characterised as the most important discriminant factor for faunal communities e.g. [9, 13–18, 19].

Various biodiversity aspects have been studied in the Mediterranean Sea e.g. [16, 19–28] but such studies are rather scarce for the eastern Mediterranean. Previous studies conducted on a Mediterranean-wide basis have provided some broad information for some eastern Mediterranean areas, such as the Aegean and Cretan Seas, averaging environmental covariates (e.g. depth, temperature) across larger geographic areas [23–28]. However, such a coarse-scale approach can mask local species-environment interactions that could provide important information about ecological processes influencing species abundance and distribution [29, 30]. Moreover, fine-scale community studies in the Aegean and Cretan Seas are rather scarce and most of them did not consider any specific community attributes. The few existing studies deal with depth-related distributional patterns of demersal megafaunal species, mostly focusing on the identification of “key-species” by depth zone [14, 31–34]. Thus, the lack of fine-scale community studies of demersal megafaunal communities in the eastern Mediterranean is a significant gap in our attempt to better understand species distribution and habitat selection patterns [35].

In the present study, a fine-scale spatial analysis of the changes in demersal megafaunal species diversity in relation to depth is presented. The analysis considers three large faunal groups, fish, cephalopods and crustaceans, and is based on data collected during the Mediterranean International Trawl Survey (MEDITS) carried out between 2005 and 2014 in the South Aegean and Cretan Seas.

## Materials and methods

### Ethic statement

Data were collected in the framework of the MEDITS project and refer to the South Aegean Sea (eastern Mediterranean). The sampling procedures followed a standardized protocol (for details see [36]), approved by international authorities (EU/DG Mare, FAO/GFCM). The specific surveys (conducted during 2005–2014) comprise a task of the Data Collection Framework for the Greek Fisheries and are accomplished with the permission of the South Aegean and Cretan Prefectures, which are responsible for the protection of the environment in the corresponding areas. The first two authors coordinate and consistently participate in the MEDITS surveys.

### Study area

The South Aegean Sea is a geographically and hydrologically complex area, with deep basins interrupted by shallower island complexes. The general large-scale water circulation pattern of the Aegean is anticlockwise. The more saline eastern Mediterranean waters flow northwards in the eastern part of the South Aegean, while the less-saline Black Sea waters flow southwards in the western part [37, 38].

**Fig 1:**
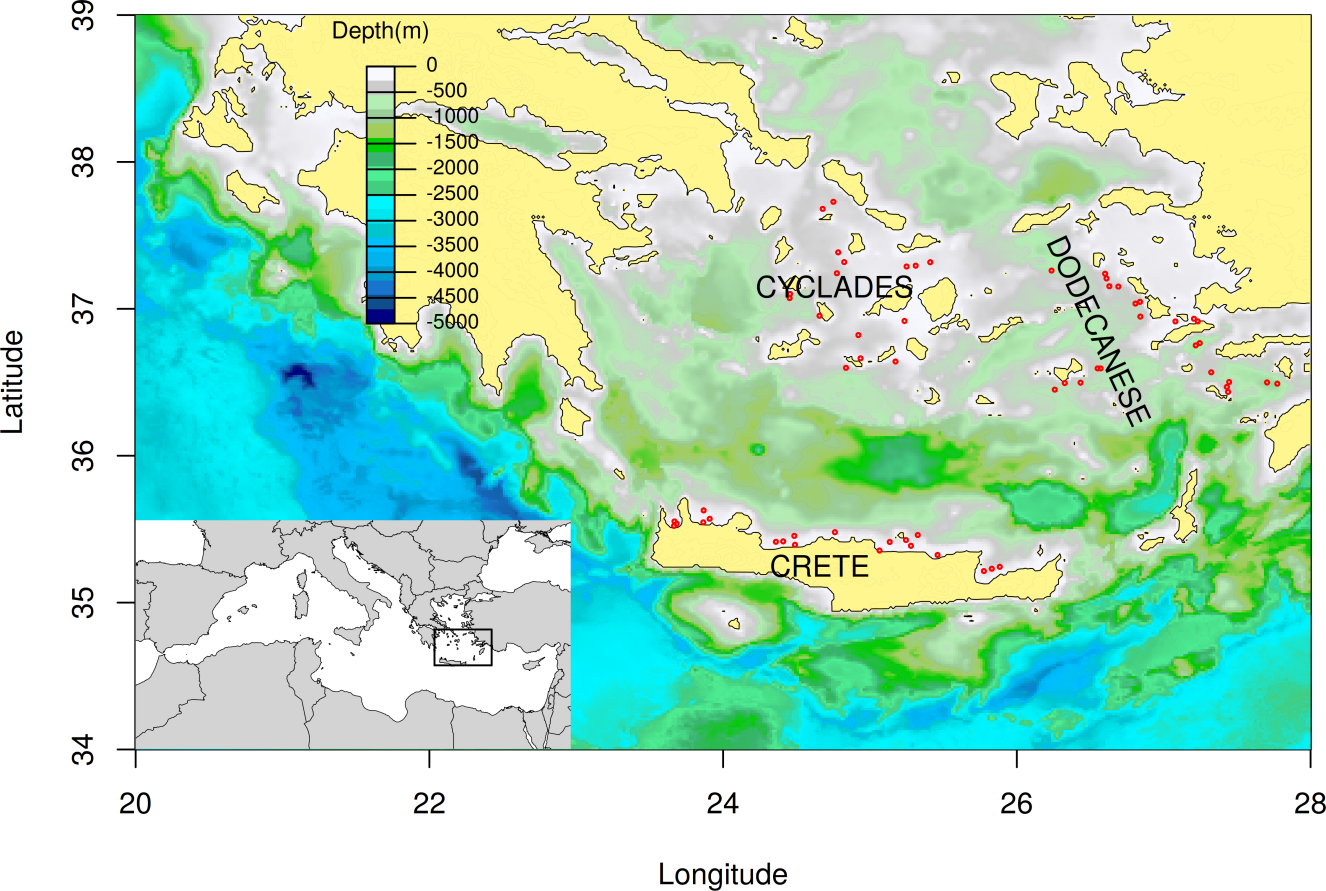
Distribution of MEDITS sampling stations (red circles) in the South Aegean and Cretan Seas.

The MEDITS sampling scheme in the South Aegean is rather discontinuous as the complex geomorphology of the area has resulted in large deep-water regions (>800 m) that are not included in the sampling protocol (Fig 1).

Taking into account the existing oceanographic characteristics and the survey sampling scheme, three subareas were considered:

1. **Crete** is an isolated island, one of the biggest in the Mediterranean, with a narrow and relatively steep continental shelf (gradient 1.5°) followed by a steep slope (2°–4°). It is surrounded by the deep Ionian, Libyan and Cretan Seas; the latter is the largest in volume and the deepest (2500 m) basin of the Aegean Sea, separating Crete from the Cycladic and Dodecanese island complexes [38]. The Cretan Sea is one of the most oligotrophic areas of the Mediterranean, with very low primary production [39] and the presence of a strong pelagic microbial loop [40], which reduces particle flux to the sediment [41]. For management purposes, Crete is considered by the General Fisheries Commission for the Mediterranean (GFCM) as a separate Geographical Subarea (GSA).
2. **Cyclades** is a complex of islands located in the middle of the Aegean Sea, comprising the Cyclades Plateau. The latter is a complex marginal platform, shallower than 250 m, with numerous islands. It separates the central from the South Aegean, and the deeper parts of the Plateau (150–250 m) are covered by sediments ranging from calcareous muddy sand to sandy mud [42]. The west edge of the Cyclades complex neighbors the Greek mainland and is influenced by the less-saline Black Sea waters [37, 43].
3. **Dodecanese** is an island complex, dispersed in the eastern part of Aegean Sea, along the coast of Minor Asia, with a high variability of depths [38] because of the wide continental shelf at the northern part, interrupted by deep waters at the southern part of the area. Most of the Dodecanese islands are situated near the large shallow bays of the Turkish coast. They are mainly influenced from the more saline eastern Mediterranean waters that flow northwards in the eastern part of Aegean. Dodecanese is separated from the Cyclades complex by deep seas, and is considered the gateway for Lessepsian species, many of which establish populations here before their westward expansion [44, 45]. Such species have been considered an important driver of diversity changes in the Mediterranean [46].

Though no specific estimates for the total fishing pressure in each subarea are available, fishing pressure can be considered higher in Cyclades, followed by Dodecanese and lower in Crete, according to the total number of registered commercial fishing vessels in each area. According to the classification of fishing areas by Sylaios et al., [47], Cyclades is characterized as a fishing area with a moderate to high fishery production, while Dodecanese and Crete are characterized as fishing areas with a low to moderate fishery production.

### Sampling

The study utilized data obtained from the MEDITS experimental surveys conducted in the South Aegean and Cretan Seas. The MEDITS survey is carried out annually in various areas of the Mediterranean Sea following a standardized protocol (see [36] for further details) and its primary goal is to monitor changes regarding the abundance and structure of the demersal megafauna community. The survey design is based on a depth-stratified random sampling scheme considering five depth strata: 10–50 m, 50–100 m, 100–200 m, 200–500 m, and 500–800 m. Sampling in the South Aegean and Cretan Seas includes a total of 61 fixed sampling stations, which are distributed from 20 to 800 m according to the surface of the depth strata (Fig 1).

For each station (haul), all fish, cephalopod and crustacean specimens were identified to the species level according to FAO species identification keys. In a few cases, specimens were identified only to the genus or family level due to difficulties in identification on board (fish: some Gobius and Myctophidae specimens; crustaceans: individuals of the genera *Munida*, *Pagurus* and *Inacus* and the family Parthenopidae; cephalopods: specimens of a few sepiolidae species and specimens in bad condition of the genus *Alloteuthis*, which were assigned to the most abundant *Alloteuthis* species caught at the station).

Consequently, the total number and biomass by species was estimated for each haul (see MEDITS manual for details [48]). In the present study, we used data from the surveys carried out during 2005–2014. Given that surveys were not conducted in 2007 and 2009–2013 in the study area, the available data refer to 2005, 2006, 2008 and 2014. Both the sampling scheme and the vessel used for the surveys were the same during the study period

### Data analysis

For each sampling station, two indices by faunistic category (fish, cephalopods and crustaceans) were estimated: (a) species richness, defined as the total number of species captured at each station and (b) the Shannon-Weaver (H΄) index (S1 Table) [49]:

$$H^{\prime }=-\sum_{i=1}^{s}p_{i}lnp_{i}$$

where, p_i_ = n_i_/N, n_i_ = number of individuals in the i_th_ species, N = total number of individuals and s = number of species

The index was computed using the “vegan” library under the R language environment [50].

A Generalized Additive Modelling (GAM) approach [51] was used to examine the effect of depth on the above indices by subarea for each faunistic category, separately. The “year” (as a factor) was also tested as a candidate predictor variable in the GAM models to account for accidental sampling inconsistencies among years due, for instance, to bad weather conditions.

Hence, the general form of the nested GAM models was:

Index=α+factor(Year)+s(Depth,by=subarea)+e

Index=α+s(Depth,by=subarea)+e

where, *a* is the intercept, *s* indicates the smoother function of the corresponding independent variable and *e* is a random error term.

The smoother function used was a penalized cubic regression spline and model fitting was accomplished using the “mgcv” library [52, 53] under the R language environment. The procedure automatically selects the degree of smoothing based on the Generalized Cross Validation (GCV) score, which is a proxy for the model predictive performance. However, to avoid dubious relationships, the model was constrained to be at maximum a quartic relationship. Hence, the maximum degrees of freedom for each smoothing term was set to 4 (i.e. k  = 5 in the GAM formulation).

Based on the diagnostic residual plots, it was found that a quasi-poisson distribution model and a log link function provided a pertinent fit to the data. The choice of the quasi-poisson distribution allowed for accounting for over-dispersion, which is common in biological data [54]. Best model selection was based on minimizing GCV scores and statistical inference was based on the 95% confidence level.

## Results

A total of 245 taxa were recorded in the 244 hauls analyzed. Out of the 245 taxa, 33 were elasmobranchs, 147 osteichthyes, 35 crustaceans and 30 cephalopods. Seven out of the eight alien fish species found were of Indo-Pacific origin, and one, *Sphoeroides pachygaster*, of Atlantic origin. Five alien species were recorded in Crete, one in Cyclades and four in Dodecanese (Table 1).

**Table 1:** Alien fish species recorded by subarea.

Origin	Alien Species	Crete	Cyclades	Dodecanese
Indo-Pacific	*Pteragogus pelycus*	**x**		
Indo-Pacific	*Siganus luridus*	**x**		
Indo-Pacific	*Siganus rivulatus*			**x**
Atlantic	*Sphoeroides pachygaster*		**x**	**x**
Indo-Pacific	*Stephanolepis diaspros*	**x**		**x**
Indo-Pacific	*Torquigener flavimaculosus*	**x**		
Indo-Pacific	*Upeneus moluccensis*	**x**		
Indo-Pacific	*Upeneus pori*	** **	** **	**x**

**Fig 2:**
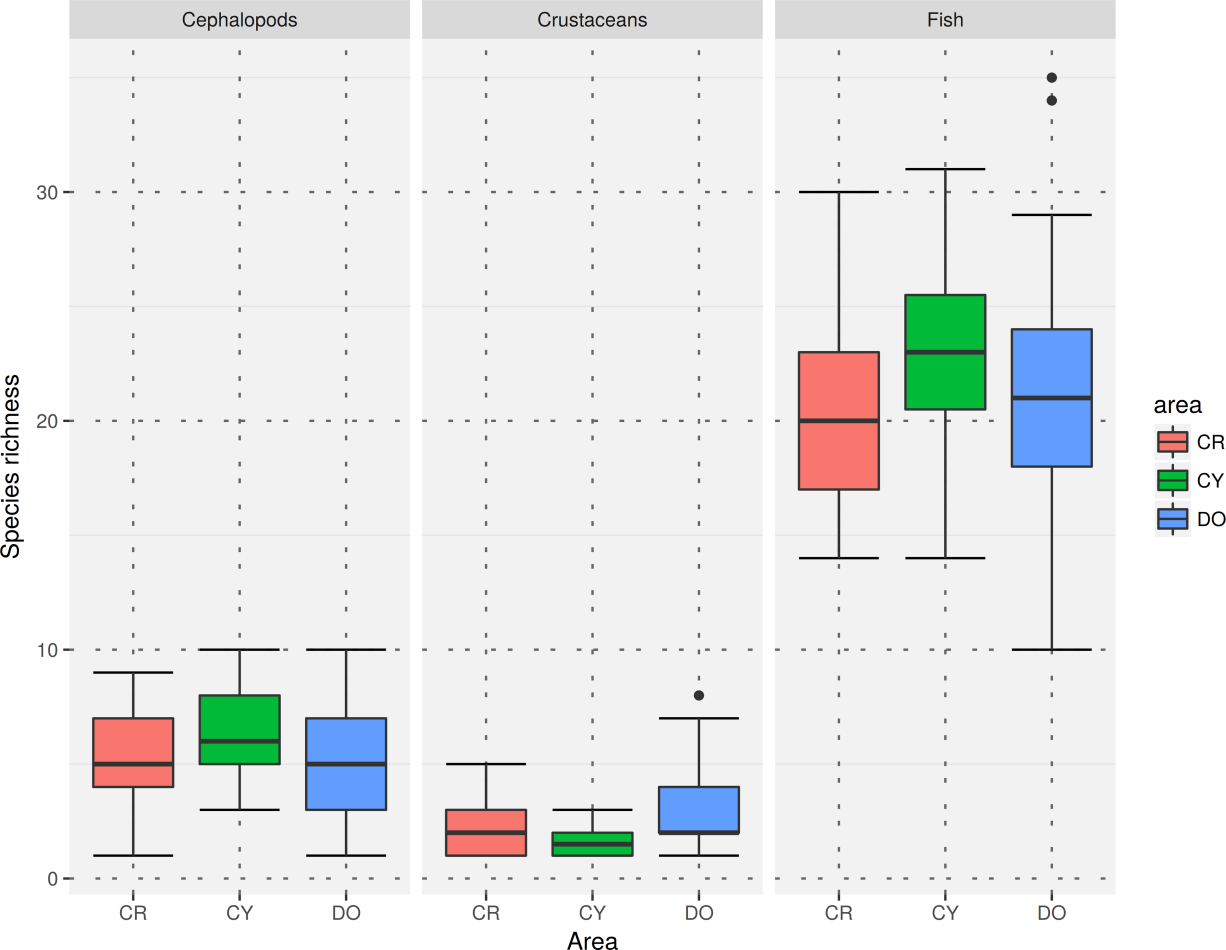
Boxplot of species richness by faunistic category and subarea (CR = Crete, CY = Cyclades, DO = Dodecanese).

**Fig 3:**
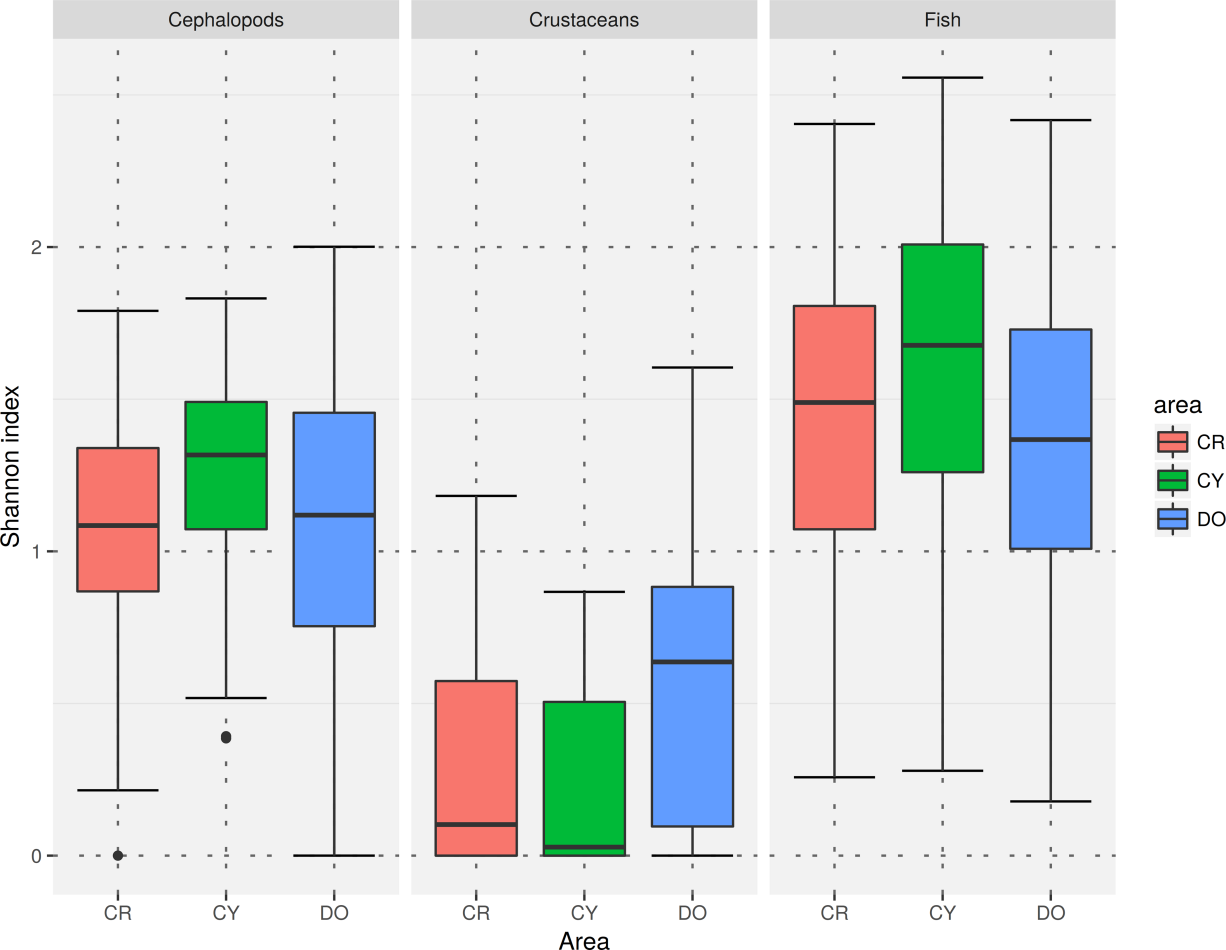
Boxplot of the Shannon-Weaver index by faunistic category and subarea (CR = Crete, CY = Cyclades, DO = Dodecanese).

For all faunistic categories, the total number of recorded taxa was higher in the Dodecanese (S2 Table). However, the median species richness by haul for fish and cephalopods were higher in Cyclades, while the highest median species richness for crustaceans was estimated in the Dodecanese. In all subareas, species richness was higher for fish and lower for crustaceans (Fig 2). Similar patterns were observed for the median Shannon-Weaver index (Fig 3).

In all cases, the inclusion of the “year” factor in the GAMs resulted in models with higher or similar GCV scores and the percentage of the deviance explained was only marginally increased (S3 Table). Thus, models without the “year” effect were considered as the best ones. The analysis of deviance of the applied GAM models on species richness indicated that the effect of depth was always significant (Table 2). Regarding the Shannon-Weaver index, depth was significant in all cases, with the exception of fish in Crete and cephalopods in Cyclades (Table 3). For both indices, the variance explained was higher in the case of cephalopods and lower for fish (Tables 2 and 3).

**Table 2:** Analysis of deviance for the applied GAM models on species richness. Numbers in brackets indicate the variance explained by each model. (CR = Crete, CY = Cyclades, DO = Dodecanese).

Model	Parameter	DF	F	P-value
***Fish***(28.75%)	*s(Depth)*:CR*s(Depth)*:CY*s(Depth)*:DO	3.523.702.95	2.283.8515.99	0.0440.004<0.001
***Cephalopods*** (61.99%)	*s(Depth)*:CR*s(Depth)*:CY*s(Depth)*:DO	3.573.253.76	13.7314.1856.86	<0.001<0.001<0.001
***Crustaceans***(49.54%)	*s(Depth)*:CR*s(Depth)*:CY*s(Depth)*:DO	11.181.89	5.1610.5855.63	0.024<0.001<0.001

**Table 3:** Analysis of deviance for the applied GAM models on the Shannon-Weaver index. Numbers in brackets indicate the variance explained by each model. (CR = Crete, CY = Cyclades, DO = Dodecanese).

Model	Parameter	DF	F	P-value
***Fish***(12.45%)	*s(Depth)*:CR*s(Depth)*:CY*s(Depth)*:DO	13.452.13	3.784.435.18	0.0530.001<0.003
***Cephalopods***(31.81%)	*s(Depth)*:CR*s(Depth)*:CY*s(Depth)*:DO	3.071.143.58	4.071.6822.43	<0.007<0.162<0.001
***Crustaceans*** (24.63%)	*s(Depth)*:CR*s(Depth)*:CY*s(Depth)*:DO	1.791.281	3.265.5931.87	0.0400.007<0.001

**Fig 4:**
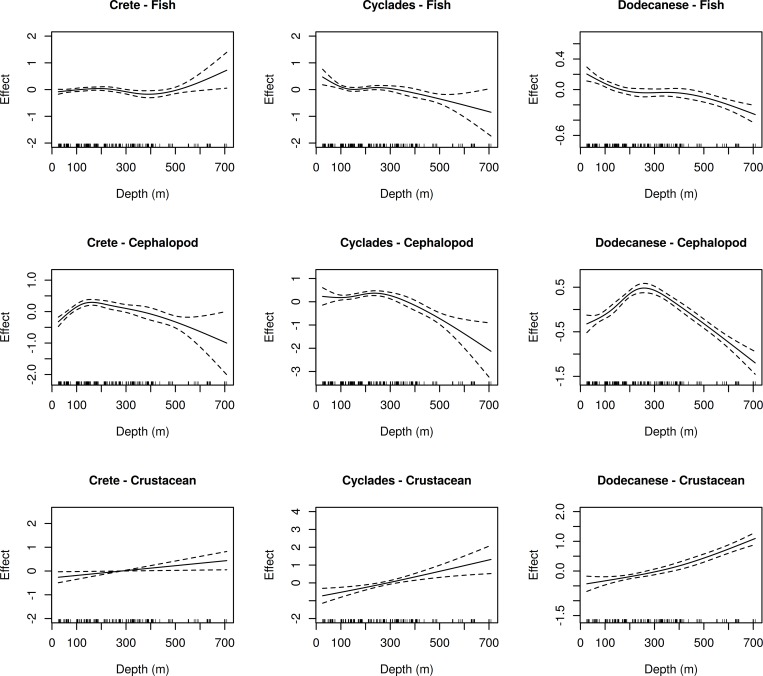
GAM-derived significant effects of depth on species richness for three megafauna groups (fish, cephalopod, crustacean) in the three subareas (Crete, Cyclades, Dodecanese). Zero line indicates mean model estimates. Broken lines indicate two standard errors and the relative density of data points is shown by the ‘‘rug” on the x-axis.

**Fig 5:**
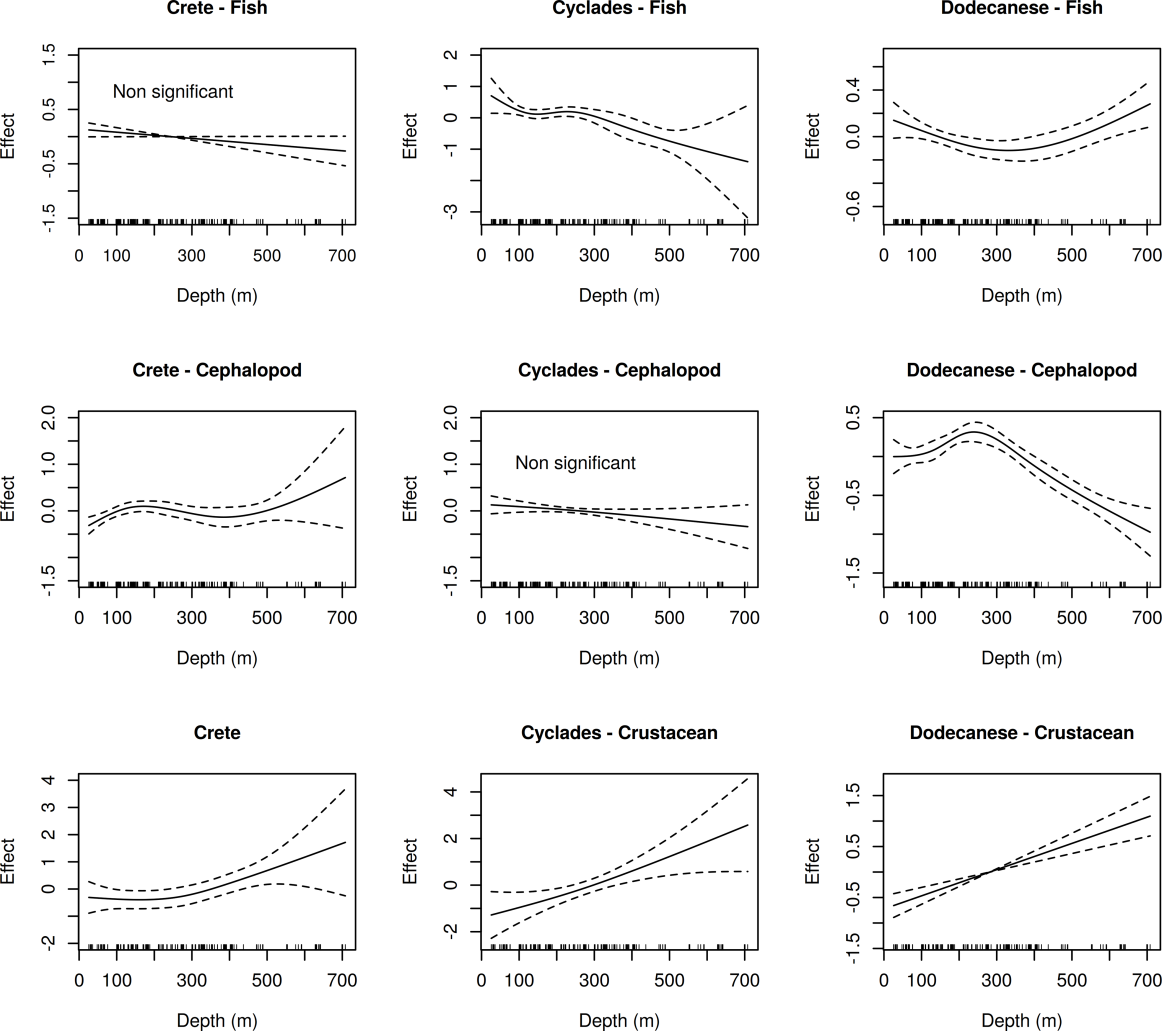
GAM-derived significant effects of depth on the Shannon-Weaver index (H΄) for three megafauna groups (fish, cephalopod, crustacean) in the three subareas (Crete, Cyclades, Dodecanese). Zero line indicates mean model estimates. Broken lines indicate two standard errors and the relative density of data points is shown by the ‘‘rug” on the x-axis.

The effect of depth (when significant) on the examined indices is presented in Figs 4 and 5.

Changes of fish species richness with depth followed similar patterns in Cyclades and Dodecanese. In both subareas, species richness decreased as depth increased. In the case of Crete, the fish species richness pattern was different, remaining rather stable down to 450 m and increasing thereafter (Fig 4).

In all subareas, changes of cephalopod species richness with depth followed a unimodal distribution pattern. However, the depths corresponding to the estimated distribution peaks were not always the same. Although Cyclades and Dodecanese had a similar peak at 250–280 m, the modal peak in Crete was observed at shallower depths (about 150 m) (Fig 4). The model estimates for crustaceans demonstrated that species richness in all subareas increased with depth (Fig 4).

Changes of the Shannon-Weaver index with depth for fish followed different patterns in Cyclades and Dodecanese. In Cyclades, H΄ decreased with an increase in depth. In Dodecanese, H΄ decreased until 300–350 m and increased thereafter. In Crete, the effect of depth on H΄ was not significant (Fig 5).

For cephalopods, the changes in H΄ with depth followed different patterns in Crete and Dodecanese. In Crete, H΄ was rather stable down to 400 m, showing an increasing trend (with relatively high confidence intervals) thereafter. In Dodecanese, H΄ showed a unimodal pattern with a peak at about 250 m. In Cyclades, the effect of depth on H΄ was not significant (Fig 5). For crustaceans, changes in H΄ with depth followed a similar pattern in all subareas, almost like that of species richness (i.e. increasing trend with depth) (Fig 5).

## Discussion

The current study adds important information to our knowledge of depth-related megafauna species diversity, by faunistic category, in three areas of the eastern Mediterranean Sea, where sampling effort is known to be much lower compared to the western Mediterranean [26]. Analysis on a fine spatial scale highlighted differences in the diversity distribution patterns among neighboring areas and faunistic categories. It must be stressed, however, that the results of the present study largely reflect only the summer conditions when the MEDITS surveys take place and thus seasonal effects cannot be taken into account.

Though species richness is assumed to be negatively influenced by low primary production and higher SST values [21, 26, 55], in the case of S. Aegean and Cretan Seas, two highly oligotrophic areas with relatively elevated SST values, a high number of taxa (245), compared to other Mediterranean areas, was recorded. In particular, the recorded numbers of elasmobranch (33), osteichthyes (147) and cephalopod (30) species were higher than the corresponding numbers of species that Colloca et al. [16] record in the western coast of Italy, (14, 128 and 26, respectively) in similar depths. The total number of fish species found (180) in the present study is close to the total number (186) recorded for the whole northern Mediterranean by Granger et al. [27]. These findings, together with previous ones [23, 24, 27], question the belief that species diversity is lower in the eastern Mediterranean [21, 26, 56]. However, the total number of cephalopod species in S. Aegean and Cretan Seas was significantly lower than the one found for the whole northern Mediterranean (58) [28], indicating a high variability in cephalopod species distribution over the Mediterranean Sea. Concerning the total number of crustacean species (35), higher numbers are recorded in different areas of W. Italy in similar depths (37 and 53) [16, 19].

Our results also indicated that alien species of Indo-Pacific origin–at least the ones available to bottom trawling–apart from Dodecanese, which is considered their gateway to the Aegean Sea [44, 45], have also extended their distribution to the Cretan Sea–corroborating findings of previous works [57–61]. However, they have not established remarkable populations in Cyclades, probably due to the influence of the Black Sea less-saline cold waters in that area [37].

Our results suggested that the effect of depth on megafauna species richness was always significant, in agreement with previous studies in various Mediterranean areas [9, 10, 16, 28, 33, 34, 62–65]. Additionally, species diversity remained stable over the study period, suggesting that environmental or anthropogenic pressure, such as climate change, fishery or touristic development, has not dramatically affected community structure during the last decade. Similar findings are reported from studies based on the MEDITS survey data in different Mediterranean areas [24, 27, 28].

Regarding the pattern of species richness distribution along the depth gradient, clear differences were detected among the three faunistic categories. For fish, several authors commonly report decreasing trends of species richness with depth in various areas of the Mediterranean [33, 34, 65]. A similar pattern was observed in the present study for fish species richness for Dodecanese and Cyclades. However, in Crete, the fish species richness-depth pattern was rather stable until 400 m depth, and increased deeper. The increasing species richness in the middle-slope areas can be related to the narrow shelf and upper slope of Crete, resulting from the strong steepness of the continental margin and the extended surface area of the lower slope [1, 49]. In an area also characterized by a steep and extended lower slope (Latio, W. Italy), Colloca et al. [16] did not find any significant trend in fish species richness with depth. However, other factors derived from anthropogenic disturbance could influence the fish community structure in Crete, such as coastal activities related to the tourist industry and development or the aggregation of fishing pressure on the narrow area of the shelf and upper slope [32].

Concerning the pattern of cephalopod and crustacean species richness along the depth gradient, our results agree with previous studies in other Mediterranean regions [16, 19, 28]. The observed constant increasing trend with depth of crustacean species richness could be attributed to specific adaptations of crustacean species, such as the great variety of feeding strategies, according to the availability of food resources. Indeed, different feeding guilds of plankton, benthos, benthopelagic, scavenging and detritus feeders (the latter mostly in bathyal depths) are described for crustacean species in other Mediterranean regions [66–68]. This variety of feeding strategies, which allows them to survive in the harsh environmental conditions of the deep sea, where food resources are limited, is most likely the main reason for the observed high species richness.

Though the interpretation of the Shannon-Weaver (Η΄) index is rather complicated [69], a direct comparison of the diversity-depth patterns derived from both measures used in the present study can provide some useful information about the community structure. For example, in the case of fish, we can assume that, for Cyclades, the species are evenly distributed within each station as both indices show similar distribution patterns. However, at the other two subareas, the observed differences between species richness and Η΄ distribution patterns along the depth gradient may indicate the existence of a few dominant species in the intermediate waters of Dodecanese and in the middle-slope areas of Crete. Comparison of the distribution patterns of both indices for cephalopods may indicate that species are quite evenly distributed, especially in the shallow waters of Dodecanese, while a few dominant species may occur on the shelf and upper slope of Crete and Cyclades. Regarding crustacean species, the similarity of species richness and Η΄ patterns with depth indicates that species are evenly distributed.

The particularly high explained deviance (61.99%) in the GAM models including cephalopod species richness as dependent variable suggests the importance of depth-related factors in structuring cephalopod communities. The observed peak at depths around the shelf break indicates that cephalopod species are favored by higher productivity [6, 70] or other specific environmental and biological factors prevailing in the shelf break zone [16]. As many cephalopods feed in the water column, it is likely that they take advantage of the segregation of planktonic, small pelagic or benthopelagic species, and/or the high diversity of epibenthic communities around the shelf break [16, 70]. In general, the exploitation of several food sources is of great importance in highly oligotrophic environments such as the South Aegean and Cretan Seas [39].

The relatively high deviance explained for crustaceans (49.54%) indicates the importance of depth-related factors in structuring crustacean communities. Effectively, sediment texture, sediment grain size and organic matter, which are directly related to depth [71, 72], play an important role in the distribution of some crustacean species e.g. [73–78]. Indeed, previous studies in the same area have reported a clear bathymetric zonation according to sediment type [50, 74].

For fish, the explained deviance was the lowest (28.75%), indicating that, besides depth-related factors, other environmental descriptors, such as habitat type and food availability, may influence community structure [39, 76]. In addition, the particular predatory and competitive abilities of fish, combined with their high mobility, allow them to develop a variety of life strategies and thus occupy larger depth ranges. For example, small-size individuals (recruits) of most species inhabit shallower depths than adults [79–83].

Differences in the species richness pattern with depth were also detected among the three subareas, with more similarities existing between Cyclades and Dodecanese (Fig 4). The decreasing trend of fish diversity with increasing depth, observed in Cyclades and Dodecanese, can be attributed to the scarcity of food resources in deeper waters that can mainly support small organisms well adapted to the conditions of deep-sea environments [84]. Previous studies in the central and eastern Mediterranean show that fish assemblages on the slope are characterized by small-sized fish species, which are mainly pelagic feeders (on mesopelagic prey), with relatively high dominance and reduced species diversity [9, 16, 33, 80]. However, our results, as mentioned before, may indicate the existence of a few dominant species in the intermediate waters of Dodecanese and the middle-slope areas of Crete.

The distinct patterns observed in Crete can be partly attributed to its geographic isolation and relatively greater distance from the mainland. In general, the distance of a marine area from the mainland is a critical factor for species diversity [15]. In addition, the specific geomorphological and oceanographic characteristics of Crete [39–41, 85, 86] have contributed to the formation of distinct species diversity patterns. Analogous differences in megafauna diversity or species assemblages between adjacent areas are also reported by other authors [12, 34, 87, 88].

The increase in fish species richness in the deeper waters of Crete could be related to the intermediate and deep layers of the Cretan Sea, which are considered a reservoir for heat, salt and dissolved oxygen [82], with the latter positively influencing species diversity [7]. In addition, the narrow, steep shelf and slope of Crete [38] may also contribute to the increase of species richness since it is known that the rate of species change is higher in steeper slopes [87]. Though fishery pressure in Crete is low [47], it is aggregated at the narrow surface of the shelf [32] and this may reduce the megafauna species diversity in the shallower waters.

Cephalopod species richness followed a unimodal distribution pattern along the depth gradient in all areas but differences were observed regarding the observed peaks. In Crete, the peak was observed at depths of around 150 m, while in Cyclades and Dodecanese, the corresponding peaks were observed in deeper waters around 250–280 m. These differences reflect the different depths of the shelf break in the corresponding areas [42, 89], indicating that the specific hydrological conditions prevailing in this transitional area (strong currents, fronts) favor cephalopod species diversity, as mentioned before. The results of a large-scale study on cephalopod diversity in the Mediterranean indicate that the peak of species richness in the whole South Aegean is between 200–300 m [28]. The present fine-scale analysis, however, allowed detecting differences among closely located areas which were masked in the large-scale study.

Regarding crustaceans, no differences were observed between subareas. Higher median values of species richness and H΄ were estimated for Cyclades regarding fish and cephalopod species. For crustaceans, the higher median values were observed in Dodecanese followed by Crete (Figs 2 and 3). Such differences can be attributed to the particular characteristics of the examined areas. For instance, the flow of the nutrient-rich Black Sea waters in the western part of Cyclades [37, 90] may influence community structure and favor species diversity. Moreover, it is likely that competition among species for niche occupation is low in the extended continental shelf of the Cyclades Plateau, thus, ranges of species can overlap, a fact that leads to increased species richness [91]. The medium-level fishing pressure in Cyclades, higher in comparison to the other study subareas [47], may also reduce species competition through reduction of the dominant species, favoring a more evenly distribution of species. Additionally, the high heterogeneity of surface sediments on the Cyclades Plateau may produce numerous different microhabitats and thus high a turnover of species, resulting in high species diversity [4, 92–94]. The surface sediments of the Cyclades Plateau consist mostly of biogenic sand (50–70%), with large amounts of coralline algae debris and terrigenous sand attributed to relict palaeobeach deposits (30–40%), and the deeper parts (150–250 m) are covered by sediments ranging from calcareous muddy sand to sandy mud [42]. Sediments in the other two areas have been described as terrigenous in origin, with coarser sediments in the coastal zone, calcareous muddy sand with small amounts of terrigenous silt and fine sand at the shelf break, and a more homogeneous fine sediment texture of hemi-pelagic deposition in the middle slope [78]. The sediment characteristics, particularly the high variability in grain size, is probably the main reason for the higher diversity values of crustaceans at Dodecanese and Crete (Fig 2).

Concluding, our results suggest differences in the species diversity–depth patterns depending on the faunal group or subarea studied. Differences were higher between Crete and the other two subareas than between Cyclades and Dodecanese, a fact that could be attributed to the specific geographical and oceanographic characteristics of this particular subarea. This finding supports the characterization by FAO/GFCM that Crete comprises a unique GSA area for fishery management. The analysis of species diversity at a fine spatial scale revealed differences masked in previously contacted large-scale studies. Thus, fine-scale analysis is important for the thorough understanding of factors affecting species diversity and, consequently, the identification of appropriate environmental conservation and management actions. Particularly, the requirements for the establishment of marine-protected areas, as mentioned in article 21 of the Marine Strategy Framework Directive (2008/56/EC), would greatly benefit from such analyses, which would provide essential information for the identification of diversity hot-spot areas.

## Supporting information

S1 Table Species density and Shannon-Weaver index by haul and faunistic category. (DOCX)

S2 Table List of all species recorded, by subarea and faunistic category. (CSV)

S3 Table GCV scores and percentage deviance explained (in parenthesis) of the nested GAM models. (XLS)
