# Supplementary material for: Comparing demersal megafaunal species diversity along the depth gradient within the South Aegean and Cretan Seas (Eastern Mediterranean)
Source: PLoS One. 2017 Sep 5;12(9):e0184241. doi: 10.1371/journal.pone.0184241 (PMC5584924; doi:10.1371/journal.pone.0184241)
Supplement: S1 Table — (DOCX) [file pone.0184241.s001.docx]

| **SPECIES** | **CRETE** | **CYCLADES** | **DODECANESE** |
| --- | --- | --- | --- |
| **Elasmobranchs** | **19** | **21** | **32** |
| *Centrophorus granulosus* |  |  | **x** |
| *Chimaera monstrosa* |  |  | **x** |
| *Dalatias licha* |  |  | **x** |
| *Dasyatis centroura* |  | **x** | **x** |
| *Dasyatis pastinaca* | **x** | **x** | **x** |
| *Dasyatis tortonesei* | **x** | **x** | **x** |
| *Dipturus batis* |  |  | **x** |
| *Dipturus oxyrinchus* | **x** | **x** | **x** |
| *Etmopterus spinax* |  |  | **x** |
| *Galeus melastomus* | **x** | **x** | **x** |
| *Heptranchias perlo* | **x** |  | **x** |
| *Leucoraja circularis* |  |  | **x** |
| *Leucoraja melitensis* |  |  | **x** |
| *Leucoraja naevus* | **x** | **x** | **x** |
| *Mustelus mustelus* |  | **x** | **x** |
| *Myliobatis aquila* | **x** |  | **x** |
| *Oxynotus centrina* | **x** | **x** | **x** |
| *Pteromylaeus bovinus* |  |  | **x** |
| *Raja asterias* | **x** | **x** | **x** |
| *Raja brachyura* | **x** | **x** | **x** |
| *Raja clavata* | **x** | **x** | **x** |
| *Raja miraletus* | **x** | **x** | **x** |
| *Raja montagui* | **x** | **x** | **x** |
| *Raja polystigma* |  | **x** | **x** |
| *Raja undulata* | **x** | **x** | **x** |
| *Scyliorhinus canicula* | **x** | **x** | **x** |
| *Scyliorhinus stellaris* |  | **x** |  |
| *Squalus acanthias* | **x** | **x** | **x** |
| *Squalus blainville* | **x** | **x** | **x** |
| *Squatina aculeata* |  |  | **x** |
| *Squatina squatina* |  |  | **x** |
| *Torpedo marmorata* | **x** | **x** | **x** |
| *Torpedo torpedo* | **x** | **x** | **x** |
| **Osteichthyes** | **107** | **82** | **116** |
| *Acantholabrus palloni* | **x** | **x** | **x** |
| *Anguilla anguilla* | **x** |  |  |
| *Anthias anthias* |  | **x** |  |
| *Argentina sphyraena* | **x** | **x** | **x** |
| *Argyropelecus hemigymnus* |  |  | **x** |
| *Arnoglossus imperialis* | **x** | **x** |  |
| *Arnoglossus laterna* | **x** | **x** | **x** |
| *Arnoglossus rueppelii* | **x** | **x** | **x** |
| *Arnoglossus thori* | **x** | **x** | **x** |
| *Aulopus filamentosus* | **x** | **x** | **x** |
| *Blennius ocellaris* | **x** | **x** | **x** |
| *Boops boops* | **x** | **x** | **x** |
| *Bothus podas* | **x** |  | **x** |
| *Brama brama* | **x** |  |  |
| *Callanthias ruber* | **x** | **x** | **x** |
| *Callionymus maculatus* | **x** |  |  |
| *Callionymus risso* | **x** |  |  |
| *Callionymus spp.* | **x** | **x** |  |
| *Capros aper* | **x** | **x** | **x** |
| *Carapus acus* | **x** | **x** |  |
| *Centracanthus cirrus* | **x** | **x** | **x** |
| *Centrolophus niger* |  |  | **x** |
| *Cepola macrophthalma* | **x** | **x** |  |
| *Ceratoscopelus maderensis* |  |  | **x** |
| *Chauliodus sloani* |  |  | **x** |
| *Chelidonichthys cuculus* | **x** | **x** | **x** |
| *Chelidonichthys lucerna* | **x** | **x** | **x** |
| *Chlorophthalmus agassizi* | **x** | **x** | **x** |
| *Chromis chromis* |  |  | **x** |
| *Citharus linguatula* | **x** | **x** | **x** |
| *Coelorinchus caelorhincus* | **x** | **x** | **x** |
| *Conger conger* | **x** | **x** | **x** |
| *Coris julis* | **x** |  | **x** |
| *Cubiceps gracilis* |  |  | **x** |
| *Deltentosteus collonianus* |  | **x** |  |
| *Deltentosteus quadrimaculatus* | **x** | **x** | **x** |
| *Dentex gibbosus* |  |  | **x** |
| *Dentex macrophthalmus* | **x** | **x** | **x** |
| *Dentex maroccanus* | **x** | **x** | **x** |
| *Diaphus holti* |  |  | **x** |
| *Diaphus metopoclampus* | **x** |  | **x** |
| *Diaphus rafinesquii* |  |  | **x** |
| *Diaphus spp.* |  |  | **x** |
| *Diplodus annularis* | **x** |  | **x** |
| *Diplodus vulgaris* |  |  | **x** |
| *Echelus myrus* | **x** | **x** | **x** |
| *Electrona risso* |  |  | **x** |
| *Engraulis encrasicolus* | **x** |  |  |
| *Epigonus denticulatus* |  |  | **x** |
| *Epinephelus aeneus* | **x** |  | **x** |
| *Eutrigla gurnardus* |  | **x** |  |
| *Evermannella balbo* |  |  | **x** |
| *Gadiculus argenteus* | **x** | **x** | **x** |
| *Gaidropsarus biscayensis* |  | **x** |  |
| *Gobius niger* | **x** |  | **x** |
| *Gobius spp.* | **x** | **x** | **x** |
| *Helicolenus dactylopterus* | **x** | **x** | **x** |
| *Hippocampus hippocampus* | **x** |  | **x** |
| *Hoplostethus mediterraneus mediterraneus* | **x** |  | **x** |
| *Hygophum benoiti* |  |  | **x** |
| *Hygophum hygomii* |  |  | **x** |
| *Hymenocephalus italicus* | **x** | **x** | **x** |
| *Labrus merula* |  |  | **x** |
| *Lampanyctus crocodilus* |  |  | **x** |
| *Lampanyctus pusillus* |  |  | **x** |
| *Lappanella fasciata* | **x** |  |  |
| *Lepidopus caudatus* | **x** | **x** | **x** |
| *Lepidorhombus boscii* | **x** | **x** | **x** |
| *Lepidorhombus whiffiagonis* | **x** | **x** | **x** |
| *Lepidotrigla cavillone* | **x** | **x** | **x** |
| *Lepidotrigla dieuzeidei* | **x** | **x** | **x** |
| *Lesueurigobius friesii* | **x** | **x** | **x** |
| *Lithognathus mormyrus* | **x** |  |  |
| *Lobianchia dofleini* |  |  | **x** |
| *Lophius budegassa* | **x** | **x** | **x** |
| *Lophius piscatorius* | **x** | **x** | **x** |
| *Macroramphosus scolopax* | **x** | **x** | **x** |
| *Maurolicus muelleri* | **x** |  | **x** |
| *Merluccius merluccius* | **x** | **x** | **x** |
| *Microchirus ocellatus* | **x** | **x** | **x** |
| *Microchirus variegatus* | **x** | **x** | **x** |
| *Micromesistius poutassou* |  | **x** | **x** |
| *Molva molva* | **x** | **x** |  |
| *Monochirus hispidus* | **x** |  |  |
| *Mullus barbatus* | **x** | **x** | **x** |
| *Mullus surmuletus* | **x** | **x** | **x** |
| *Myctophidae* |  |  | **x** |
| *Nettastoma melanurum* |  |  | **x** |
| *Nezumia sclerorhynchus* |  |  | **x** |
| *Notacanthus bonaparte* |  |  | **x** |
| *Pagellus acarne* | **x** | **x** | **x** |
| *Pagellus bogaraveo* | **x** | **x** | **x** |
| *Pagellus erythrinus* | **x** | **x** | **x** |
| *Pagrus pagrus* | **x** |  | **x** |
| *Paralepis speciosa* |  |  | **x** |
| *Peristedion cataphractum* | **x** | **x** | **x** |
| *Phycis blennoides* | **x** | **x** | **x** |
| *Phycis phycis* |  | **x** |  |
| *Pteragogus pelycus* | **x** |  |  |
| *Sardina pilchardus* | **x** | **x** |  |
| *Sardinella aurita* | **x** |  |  |
| *Scomber colias* | **x** | **x** | **x** |
| *Scomber scombrus* |  | **x** |  |
| *Scorpaena elongata* | **x** | **x** | **x** |
| *Scorpaena notata* | **x** | **x** | **x** |
| *Scorpaena porcus* | **x** | **x** | **x** |
| *Scorpaena scrofa* | **x** | **x** | **x** |
| *Serranus cabrilla* | **x** | **x** | **x** |
| *Serranus hepatus* | **x** | **x** | **x** |
| *Serranus scriba* |  |  | **x** |
| *Siganus luridus* | **x** |  |  |
| *Siganus rivulatus* |  |  | **x** |
| *Solea solea* | **x** |  |  |
| *Sparisoma cretense* | **x** |  | **x** |
| *Sparus aurata* |  |  | **x** |
| *Sphoeroides pachygaster* |  | **x** | **x** |
| *Sphyraena sphyraena* | **x** |  |  |
| *Spicara flexuosa* | **x** | **x** | **x** |
| *Spicara maena* | **x** | **x** | **x** |
| *Spicara smaris* | **x** | **x** | **x** |
| *Spondyliosoma cantharus* | **x** | **x** | **x** |
| *Stephanolepis diaspros* | **x** |  | **x** |
| *Symphodus cinereus* | **x** |  | **x** |
| *Symphodus rostratus* | **x** |  |  |
| *Symphodus tinca* | **x** |  | **x** |
| *Symphurus ligulatus* |  |  | **x** |
| *Symphurus nigrescens* | **x** | **x** |  |
| *Synchiropus phaeton* | **x** | **x** | **x** |
| *Syngnathus acus* | **x** |  | **x** |
| *Syngnathus spp.* |  |  | **x** |
| *Syngnathus typhle* | **x** |  | **x** |
| *Synodus saurus* | **x** | **x** | **x** |
| *Torquigener flavimaculosus* | **x** |  |  |
| *Trachinus araneus* |  | **x** | **x** |
| *Trachinus draco* | **x** | **x** | **x** |
| *Trachinus radiatus* | **x** | **x** | **x** |
| *Trachurus mediterraneus* | **x** |  |  |
| *Trachurus picturatus* | **x** | **x** | **x** |
| *Trachurus trachurus* | **x** | **x** | **x** |
| *Trigla lyra* | **x** | **x** | **x** |
| *Trigloporus lastoviza* | **x** | **x** | **x** |
| *Trisopterus capelanus* | **x** | **x** | **x** |
| *Upeneus moluccensis* | **x** |  |  |
| *Upeneus pori* |  |  | **x** |
| *Uranoscopus scaber* | **x** | **x** | **x** |
| *Vinciguerria attenuata* | **x** |  |  |
| *Zeus faber* | **x** | **x** | **x** |
| **Crustacean** | **21** | **15** | **25** |
| *Aegaeon cataphractus* | **x** | **x** |  |
| *Aegaeon lacazei* | **x** |  |  |
| *Aristaeomorpha foliacea* |  |  | **x** |
| *Aristeus antennatus* |  |  | **x** |
| *Bathynectes maravigna* |  |  | **x** |
| *Calappa granulata* | **x** | **x** | **x** |
| *Chlorotocus crassicornis* |  | **x** |  |
| *Dromia personata* |  | **x** |  |
| *Eriphia verrucosa* | **x** |  | **x** |
| *Inachus spp.* | **x** |  |  |
| *Latreillia* | **x** |  | **x** |
| *Liocarcinus depurator* | **x** | **x** | **x** |
| *Liocarcinus spp.* | **x** | **x** | **x** |
| *Macropipus tuberculatus* | **x** |  |  |
| *Maja squinado* | **x** | **x** | **x** |
| *Munida spp.* | **x** | **x** | **x** |
| *Nephrops norvegicus* | **x** | **x** | **x** |
| *Oplophoridae* |  |  | **x** |
| *Pagurus spp.* | **x** | **x** | **x** |
| *Palinurus elephas* | **x** |  |  |
| *Parapenaeus longirostris* | **x** | **x** | **x** |
| *Parthenopidae* | **x** | **x** |  |
| *Pasiphaea multidentata* |  |  | **x** |
| *Pasiphaea sivado* | **x** |  | **x** |
| *Plesionika acanthonotus* |  |  | **x** |
| *Plesionika edwardsii* | **x** | **x** | **x** |
| *Plesionika heterocarpus* | **x** | **x** | **x** |
| *Plesionika martia* | **x** |  | **x** |
| *Plesionika narval* |  |  | **x** |
| *Plesionika spp.* |  | **x** |  |
| *Polycheles typhlops* |  |  | **x** |
| *Sergestidae* |  |  | **x** |
| *Sicyonia carinata* | **x** |  |  |
| *Solenocera membranacea* |  |  | **x** |
| *Squilla mantis* | **x** | **x** | **x** |
| **Cephalopod** | **21** | **20** | **25** |
| *Alloteuthis media* | **x** | **x** | **x** |
| *Alloteuthis subulata* | **x** | **x** |  |
| *Ancistrocheirus lesueurii* |  |  | **x** |
| *Ancistroteuthis lichtensteinii* |  |  | **x** |
| *Brachioteuthis riisei* |  |  | **x** |
| *Eledone cirrhosa* | **x** | **x** | **x** |
| *Eledone moschata* | **x** | **x** | **x** |
| *Heteroteuthis dispar* |  |  | **x** |
| *Histioteuthis bonnellii* |  |  | **x** |
| *Histioteuthis reversa* |  |  | **x** |
| *Illex coindetii* | **x** | **x** | **x** |
| *Loligo forbesi* | **x** | **x** | **x** |
| *Loligo vulgaris* | **x** | **x** | **x** |
| *Neorossia caroli* |  | **x** | **x** |
| *Octopoteuthis sicula* |  |  | **x** |
| *Octopus vulgaris* | **x** | **x** | **x** |
| *Onychoteuthis banksii* |  |  | **x** |
| *Pteroctopus tetracirrhus* | **x** |  | **x** |
| *Rondeletiola minor* | **x** | **x** |  |
| *Rossia macrosoma* | **x** | **x** | **x** |
| *Scaeurgus unicirrhus* | **x** | **x** | **x** |
| *Sepia elegans* | **x** | **x** | **x** |
| *Sepia officinalis* | **x** | **x** | **x** |
| *Sepia orbignyana* | **x** | **x** | **x** |
| *Sepietta oweniana* | **x** | **x** |  |
| *Sepiola rondeleti* | **x** |  |  |
| *Sepiola spp.* | **x** | **x** |  |
| *Sepiolidae* | **x** | **x** | **x** |
| *Todarodes sagittatus* | **x** | **x** | **x** |
| *Todaropsis eblanae* | **x** | **x** | **x** |
| **TOTAL** | **168** | **138** | **198** |
